# Supplementary material for: Machine Learning for Multi-Omics Characterization of Blood Cancers: A Systematic Review
Source: Cells. 2025 Sep 4;14(17):1385. doi: 10.3390/cells14171385 (PMC12427946; doi:10.3390/cells14171385)
Supplement: Supplementary file 1 [file cells-14-01385-s001.zip › cells-3745830-supplementary.pdf]

Supplementary Material

Supplementary Materials S1: PRISMA 2020 Checklist

**Section and Topic**	**Item #**	**Checklist Item**
**TITLE**		
Title	1	Identify the report as a systematic review
**ABSTRACT**		
Abstract	2	See the PRISMA 2020 for Abstracts checklist
**INTRODUCTION**		
Rationale	3	Describe the rationale for the review
Objectives	4	Provide an explicit statement of the objective(s)
**METHODS**		
Eligibility criteria	5	Specify the inclusion and exclusion criteria
Information sources	6	Specify all databases, registers, websites searched
Search strategy	7	Present the full search strategies for all databases
Selection process	8	Describe the methods used to decide whether a study met the inclusion criteria
Data collection process	9	Describe the methods used to collect data from reports
Data items	10a	List and define all outcomes for which data were sought
Study risk of bias assessment	11	Describe the methods used to assess risk of bias
Effect measures	12	Specify for each outcome the effect measure used
Synthesis methods	13a	Describe the processes used to decide which studies were eligible for each synthesis
Reporting bias assessment	14	Describe any methods used to assess risk of bias due to missing results
Certainty assessment	15	Describe any methods used to assess certainty of the body of evidence
**RESULTS**		
Study selection	16a	Describe the results of the search and selection process
Study characteristics	17	Cite each included study and present its characteristics
Risk of bias in studies	18	Present assessments of risk of bias for each included study
Results of individual studies	19	Present the results of each study for each outcome
Results of syntheses	20a	Present results of each synthesis conducted
Reporting biases	21	Present assessments of risk of bias due to missing results
Certainty of evidence	22	Present assessments of certainty for each important outcome

**DISCUSSION**		
Discussion	23a	Provide a general interpretation of the results
Limitations	23b	Discuss limitations of the evidence included in the review
Conclusions	23c	Provide a general interpretation of the results
**OTHER INFORMATION**		
Registration and protocol	24a	Provide registration information, including registration number
Support	25	Describe sources of financial or other support for the review
Competing interests	26	Declare any competing interests of review authors

Supplementary Materials S2: Search Strategies for All Databases

B.1 PubMed/MEDLINE Search Strategy

#1 "artificial intelligence"[MeSH Terms] OR "machine learning"[MeSH Terms] #2 "deep learning"[Title/Abstract] OR "neural network*"[Title/Abstract] #3 "support vector machine*"[Title/Abstract] OR "random forest*"[Title/Abstract] #4 "ensemble method*"[Title/Abstract] OR "decision tree*"[Title/Abstract] #5 #1 OR #2 OR #3 OR #4 #6 "hematologic neoplasms"[MeSH Terms] OR "leukemia"[MeSH Terms] #7 "lymphoma"[MeSH Terms] OR "multiple myeloma"[MeSH Terms] #8 "hematological malignancies"[Title/Abstract] OR "blood cancer*"[Title/Abstract] #9 #6 OR #7 OR #8 #10 "genomics"[MeSH Terms] OR "transcriptomics"[Title/Abstract] #11 "proteomics"[MeSH Terms] OR "metabolomics"[MeSH Terms] #12 "multi-omics"[Title/Abstract] OR "molecular characterization"[Title/Abstract] #13 "biomarker*"[Title/Abstract] OR "molecular signature*"[Title/Abstract] #14 #10 OR #11 OR #12 OR #13 #15 #5 AND #9 AND #14 #16 #15 AND ("2015/01/01"[Date - Publication] : "2024/12/31"[Date - Publication]) #17 #16 AND English[Language]

B.2 Embase Search Strategy

#1 'artificial intelligence'/exp OR 'machine learning'/exp #2 'deep learning':ti,ab OR 'neural network*':ti,ab #3 'support vector machine*':ti,ab OR 'random forest*':ti,ab #4 'ensemble method*':ti,ab OR 'decision tree*':ti,ab #5 #1 OR #2 OR #3 OR #4 #6 'hematologic malignancy'/exp OR 'leukemia'/exp #7 'lymphoma'/exp OR 'multiple myeloma'/exp #8 'hematological malignancies':ti,ab OR 'blood cancer*':ti,ab #9 #6 OR #7 OR #8 #10 'genomics'/exp OR 'transcriptomics':ti,ab #11 'proteomics'/exp OR 'metabolomics'/exp #12 'multi-omics':ti,ab OR 'molecular characterization':ti,ab #13 'biomarker*':ti,ab OR 'molecular signature*':ti,ab #14 #10 OR #11 OR #12 OR #13 #15 #5 AND #9 AND #14 #16 #15 AND [2015-2024]/py AND [english]/lim

B.3 IEEE Xplore Search Strategy

((("All Metadata":"artificial intelligence") OR ("All Metadata":"machine learning") OR ("All Metadata":"deep learning") OR ("All Metadata":"neural network")) AND (("All Metadata":"hematological malignancies") OR ("All Metadata":"leukemia") OR ("All Metadata":"lymphoma") OR ("All Metadata":"multiple myeloma")) AND (("All Metadata":"genomics") OR ("All Metadata":"transcriptomics") OR ("All Metadata":"proteomics") OR ("All Metadata":"multi-omics"))) Filters Applied: Publication Year: 2015-2024

B.4 Web of Science Search Strategy

TS=(("artificial intelligence" OR "machine learning" OR "deep learning" OR "neural network*" OR "support vector machine*" OR "random forest*") AND ("hematological malignancies" OR "leukemia" OR "lymphoma" OR "multiple myeloma" OR "blood cancer*") AND

("genomics" OR "transcriptomics" OR "proteomics" OR "metabolomics" OR "multi-omics" OR "molecular characterization")) Timespan: 2015-2024 Language: English Document Types: Article, Review

Supplementary Materials S3: Data Extraction Form

C.1 Study Identification

First Author
Publication Year
Journal
Country/Region
Study Design
Registration Number (if applicable)

C.2 Population Characteristics

Sample Size (training/validation/test sets)
Hematological Malignancy Type
Disease Subtype/Stage
Age (mean/median, range)
Sex Distribution
Ethnicity/Race (if reported)
Treatment Status
Follow-up Duration

C.3 Omics Data Characteristics

Data Types (genomics, transcriptomics, proteomics, metabolomics, epigenomics)
Platform/Technology Used
Number of Features
Data Sources
Public Dataset Usage
Data Quality Control Measures
Preprocessing Methods
Batch Effect Correction

C.4 AI/ML Methodology

Algorithm Type(s)
Feature Selection Methods
Hyperparameter Optimization
Cross-validation Strategy
Model Development Process
Software/Tools Used
Computational Resources
Code Availability

C.5 Performance Metrics

Primary Outcome Measure
Sensitivity
Specificity
Accuracy
AUC/C-index
Positive Predictive Value
Negative Predictive Value
F1-Score
Confidence Intervals
Statistical Significance Tests

C.6 Validation and Generalizability

Internal Validation Method

External Validation Performed (Y/N)
External Validation Dataset(s)
Multi-center Validation
Temporal Validation
Cross-population Validation
Performance Degradation in External Sets

C.7 Explainability and Interpretability

Explainability Method Used
Feature Importance Reported
Biological Interpretation Provided
Pathway Analysis Conducted
Mechanistic Insights Generated
Clinical Actionability

C.8 Quality and Risk of Bias

QUADAS-AI Domain Ratings
Risk of Overfitting
Data Leakage Prevention
Selection Bias
Reporting Completeness
Conflict of Interest

C.9 Clinical Translation

Clinical Utility Assessment
Decision Curve Analysis
Cost-effectiveness Evaluation
Implementation Considerations
Regulatory Pathway Discussion
Clinical Trial Integration

C.10 Ethical Considerations

IRB/Ethics Approval
Informed Consent
Data Privacy Measures
Bias Assessment
Fairness Evaluation
Population Diversity
Data Sharing Policies

Supplementary Materials S4: Quality Assessment Tool (Modified QUADAS-AI)

D.1 Risk of Bias Assessment

Domain 1: Patient Selection

Signaling Questions:
Was a consecutive or random sample of patients enrolled?
Was a case-control design avoided?
Did the study avoid inappropriate exclusions?
Was the patient spectrum representative of clinical practice?
Risk of Bias: Low/High/Unclear

Domain 2: Index Test (AI/ML Model)

Signaling Questions:
Were the AI/ML model development and validation procedures clearly described?

Was the model performance assessment conducted without knowledge of the reference standard results?
Were pre-specified thresholds used?
Was overfitting adequately addressed?
Was data leakage prevented?
Risk of Bias: Low/High/Unclear

Domain 3: Reference Standard

Signaling Questions:
Is the reference standard likely to correctly classify the target condition?
Were the reference standard results interpreted without knowledge of the results of the index test?
Was the reference standard applied consistently?
Risk of Bias: Low/High/Unclear

Domain 4: Flow and Timing

Signaling Questions:
Was there an appropriate interval between index test and reference standard?
Did all patients receive the same reference standard?
Were all patients included in the analysis?
Were withdrawals from the study explained?
Risk of Bias: Low/High/Unclear

Domain 5: AI-Specific Considerations

Signaling Questions:
Was the training dataset independent from the test dataset?
Was hyperparameter tuning performed using appropriate validation strategies?
Were measures taken to prevent data leakage?
Was model complexity appropriate for the dataset size?
Were appropriate statistical methods used for performance evaluation?
Risk of Bias: Low/High/Unclear

D.2 Applicability Assessment

Domain 1: Patient Selection Applicability

Questions:
Are there concerns that the included patients do not match the review question?
Is the patient spectrum representative of the intended use population?
Applicability Concerns: Low/High/Unclear

Domain 2: Index Test Applicability

Questions:

Are there concerns that the index test, its conduct, or interpretation differ from the review question?
Is the AI/ML implementation feasible in clinical practice?
Applicability Concerns: Low/High/Unclear

Domain 3: Reference Standard Applicability

Questions:
Are there concerns that the target condition as defined by the reference standard does not match the review question?
Is the reference standard appropriate for clinical practice?
Applicability Concerns: Low/High/Unclear
